# Human astrocytes in the diseased brain

**DOI:** 10.1016/j.brainresbull.2017.02.001

**Published:** 2018-01

**Authors:** Elena Dossi, Flora Vasile, Nathalie Rouach

**Affiliations:** Neuroglial Interactions in Cerebral Physiopathology, Center for Interdisciplinary Research in Biology, Collège de France, CNRS UMR 7241, INSERM U1050, Labex Memolife, PSL Research University, Paris, France

**Keywords:** Aβ, amyloid Beta, AQP, aquaporin, AD, Alzheimer’s disease, CNS, central nervous system, Cx, connexin, DS, Down syndrome, GJ, gap junction, GFAP, glial fibrillary acidic protein, GS, glutamine synthetase, HS, hippocampal sclerosis, IP3R2, inositol triphosphate receptor 2, MDD, major depressive disorder, MTLE, mesial temporal lobe epilepsy, mGluR5, metabotropic glutamate receptor 5, Astrocytes, Humans, Brain, Pathology

## Abstract

Astrocytes are key active elements of the brain that contribute to information processing. They not only provide neurons with metabolic and structural support, but also regulate neurogenesis and brain wiring. Furthermore, astrocytes modulate synaptic activity and plasticity in part by controlling the extracellular space volume, as well as ion and neurotransmitter homeostasis. These findings, together with the discovery that human astrocytes display contrasting characteristics with their rodent counterparts, point to a role for astrocytes in higher cognitive functions. Dysfunction of astrocytes can thereby induce major alterations in neuronal functions, contributing to the pathogenesis of several brain disorders. In this review we summarize the current knowledge on the structural and functional alterations occurring in astrocytes from the human brain in pathological conditions such as epilepsy, primary tumours, Alzheimer’s disease, major depressive disorder and Down syndrome. Compelling evidence thus shows that dysregulations of astrocyte functions and interplay with neurons contribute to the development and progression of various neurological diseases. Targeting astrocytes is thus a promising alternative approach that could contribute to the development of novel and effective therapies to treat brain disorders.

## Highlights

• Astrocytes are active dynamic signalling players of the brain.
• Dysfunctions of astrocytes can contribute to the pathogenesis of brain disorders.
• A common pathological hallmark of several CNS diseases is reactive astrogliosis.
• Targeting astrocytes represent an alternative approach to develop new therapies.

## 1 Introduction

Astrocytes are active dynamic signalling players of the central nervous system (CNS). Over the past 25 years it has become clear that astrocytes participate to a variety of essential physiological processes in the healthy brain. Indeed, far from being merely passive cells providing structural support to neurons, astrocytes are now viewed as crucial active and dynamic elements of the brain circuitry: they participate in formation and maturation of synapses, receptor trafficking, control of the homeostasis of ions and energy metabolites and clearance of neurotransmitters. They also regulate the extracellular space volume and modulate the moment-to-moment synaptic plasticity (Araque et al., 2014, Dallérac and Rouach, 2016). Many studies have shown their contribution to information processing and memory formation in the brain, thereby pointing to a role of astrocytes in higher integrated brain functions. Dynamic bidirectional signalling between astrocytes and neurons has mainly been reported in experimental animal models. Recent data however show that such reciprocal signalling also occurs in the human brain. Astrocytes from human brain tissue indeed exhibit Ca^2+^-based “intrinsic excitability” and can respond to synaptically-released neurotransmitters (Navarrete et al., 2012). Furthermore, morphological, genomic and functional studies have revealed that human astrocytes display specific characteristics compared to the rodent counterpart (Miller et al., 2010, Oberheim et al., 2006, Oberheim et al., 2009, Zhang et al., 2016, Zheng et al., 2015). Human astrocytes display a remarkable morphological diversity according to cortical layers, being larger and more complex than those of rodents; furthermore, they exhibit a high expression of proteins involved in Ca^2+^ signalling and propagate Ca^2+^ waves at much faster velocities than their rodent counterparts (Bazargani and Attwell, 2016, Oberheim et al., 2009). Altogether, these findings support the idea that in the human brain, astrocytes may play a crucial role underlying higher cognitive functions. Alterations in astrocyte physiological roles have thus been hypothesized to contribute to cerebral pathology. Indeed, as early as in the 19^th^ century, several neuropathologists such as Alzheimer, Fromman and Nissl, already envisioned a role for glia in brain diseases. Nonetheless, since the beginning of the 20^th^ century the concept that neurological diseases result primarily from neuronal dysfunction dominated. However, this neurocentric paradigm did not systematically lead to prominent advances in therapies for brain diseases. Such diseases indeed still remain the most complicated to understand and treat. Growing evidence from analysis of post-mortem or surgically resected human tissues and from animal models of CNS pathologies indicate that astroglial dysfunctions contribute to the pathogenesis of several neurological and psychiatric disorders (Halassa et al., 2007, Rossi and Volterra, 2009).

In this review we focus on human-specific astroglial changes in some frequent neurological disorders, such as epilepsy, brain tumours, Alzheimer’s disease, major depressive disorder and Down syndrome.

## 2 Astrogliosis as a hallmark of brain diseases

**Fig. 1:**
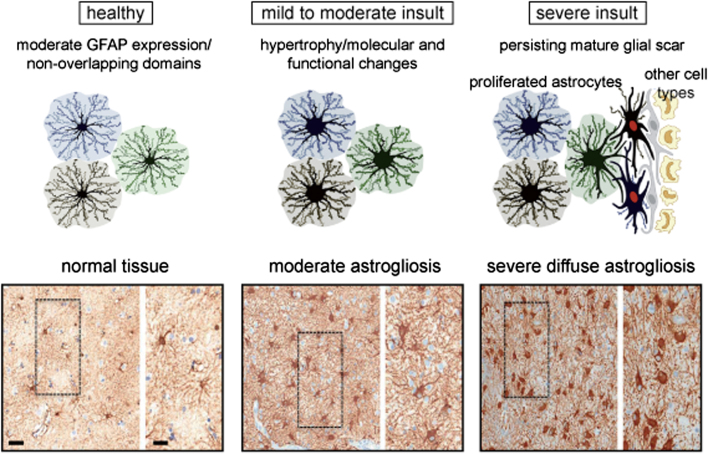
**Astrogliosis in pathological human brain**. Top, schematic representation of different gradation of astrogliosis depending on the gravity of the insult. Bottom, astrocyte morphology in normal tissue from a human autopsy specimen far from a lesion (left), and in presence of moderate (middle) and severe diffuse reactive astrogliosis (right). For each condition, a brightfield immunochemistry for GFAP counterstained with haematoxylin is shown on the left, and an enlarged view of the boxed areas on the right. Scale bars: left, 50 μm; right, 20 μm. [upper part modified from (Sofroniew, 2009); bottom part from (Sofroniew and Vinters, 2010)].

A common feature and pathological hallmark of several CNS diseases is reactive astrogliosis (Fig. 1). It consists of a finely graded continuum of molecular, cellular and functional changes in astrocytes in response to CNS injuries; these alterations vary according to the severity of the disease (Anderson et al., 2014, Eddleston and Mucke, 1993) and are regulated through inter- and intracellular signalling molecules in a context-specific manner (Sofroniew, 2009). In mild or moderate astrogliosis, which is generally associated with mild trauma or located in areas at a certain distance from CNS lesions, astrocytic proliferation is almost absent. Variable increased glial fibrillary acidic protein (GFAP) expression has also been observed, together with cell body and process hypertrophy, which is not altering astrocyte organization into individual distinct domains (Wilhelmsson et al., 2006). Furthermore, other proteins are up-regulated in reactive astrocytes, such as copper-zinc superoxide dismutase, glutathione peroxidase or metallothionein. Moderate astrogliosis also results in expression of inducible nitric oxide synthase and release of trophic factors and cytokines, including tumour necrosis factors α and β, interleukins and interferons (Chen and Swanson, 2003). In mild or moderate forms, reactive astrogliosis exhibits the potential for resolution, if the initial triggering insult resolves or is removed; in this case, cells return to a condition similar to that observed in healthy tissue (Sofroniew, 2009). On the contrary, near focal lesions, infections or neurodegenerative areas severe diffuse astrogliosis is characterized by enhanced astrocytic proliferation. Molecular factors promoting proliferation of reactive astrocytes are not completely characterized, but a role for epidermal growth factor, fibroblast growth factor, endothelin 1, ATP, lipopolysaccharide and nitric oxide has been identified (Gadea et al., 2008, Levison et al., 2000, Neary and Zimmermann, 2009, Sofroniew and Vinters, 2010). This enhanced astrocytic proliferation causes intermingling and overlapping of neighbouring astrocytic processes, which disrupts individual astrocyte domains. In some cases, this potent astrocytic reaction can drive the formation of a compact glial scar (Fig. 1). Such scar is characterized by astrocyte interaction with different cell types and is mainly formed along the borders of severe tissue damage, necrosis, tumours, chronic neurodegeneration, infection or inflammatory infiltration (Sofroniew, 2009, Sofroniew and Vinters, 2010). These structural changes are long-lasting and persist after the resolution of the triggering insult (Sofroniew, 2009). Moreover, mature glial scars act as barriers to inflammatory cells to protect surrounding healthy tissue from nearby areas of intense inflammation. Reactive astrocytes can also protect CNS cells and tissue by uptaking excitotoxic glutamate, producing glutathione against oxidative stress, degrading amyloid β peptides, regulating extracellular space volume and ion balance, facilitating blood brain barrier repair and regulating CNS inflammation. Nevertheless, growing evidence also shows that reactive astrocytes can contribute to or be the primary source of CNS physiopathology. Reactive astrocytes from glial scars can indeed synthesize collagen and sulphate proteoglycans, which prevent axon regeneration (Chen and Swanson, 2003). In addition, alteration of the physiological functions of astrocytes resulting from genetic mutations contribute to brain disorders such as Alexander’s disease and amyotrophic lateral sclerosis (Brenner et al., 2001, Nagai et al., 2007). These opposite effects of reactive astrocytes thus point to a dual function of astrogliosis (Sofroniew, 2009, Sofroniew and Vinters, 2010).

## 3 Epilepsy

**Fig. 2:**
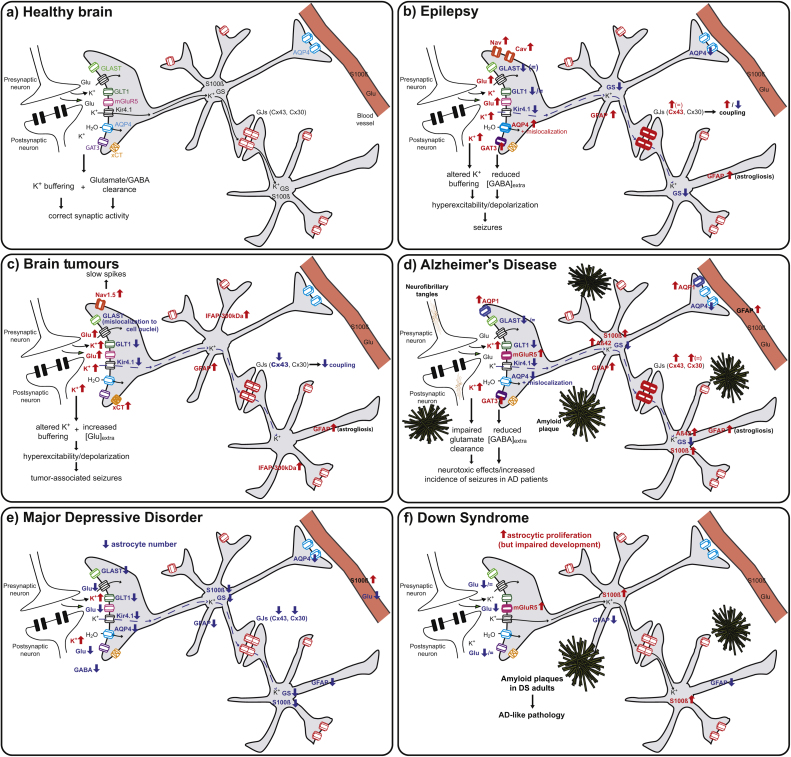
**Alterations of astrocytes in CNS disorders**. (a) Schematic representation of astrocyte-mediated regulation of synaptic activity in the healthy brain. (b–f) Changes of astrocytic receptors, transporters, ion channels and intracellular proteins in epilepsy (b), brain tumours (c), Alzheimer’s disease (d), major depressive disorder (**e**) and Down syndrome (f). Increases of expression/levels are indicated in red, decreases in blue. [AQP4: aquaporin 4; Cav: voltage-gated Ca^2+^ channels; Cx43 and Cx30: connexins 43 and 30; GAT3: GABA transporter 3; GFAP: glial fibrillary acidic protein; GLAST and GLT1: glutamate transporters; Glu: glutamate; GS: glutamine synthetase; GJs: gap junctions; Kir4.1: inwardly-rectifying K^+^ channels; mGluR5: metabotropic glutamate receptor 5; Nav: voltage-gated Na^+^ channels; xCT: cysteine-glutamate system].

Epilepsy is one of the most prevalent neurological diseases affecting 1% of the world population (World Health Organisation, 2016, http://www.who.int/en/). It is characterized by repetitively recurrent seizures, which disrupt normal brain functions and can damage the brain and worsen pre-existing neurological deficits. Contrary to the traditional view assuming that epileptic activity is generated exclusively in and by neurons, an astrocytic basis for epilepsy has been proposed (Tian et al., 2005). Moreover, investigations on specimens from mesial temporal lobe epilepsy (MTLE) patients have identified changes in astrocytic channels and receptors (Fig. 2a–b), thus suggesting that astrocyte dysfunction can participate in hyper-excitation, neurotoxicity and seizure spreading, in addition to established neurogenic mechanisms.

### 3.1 Epilepsy-associated astrogliosis

Reactive astrogliosis is present in almost all forms of epilepsy, but it is most notable in presence of hippocampal sclerosis (HS), which is often associated with MTLE and other epilepsy syndromes (Thom, 2014). Indeed, besides a severe loss of principal neurons observed in CA1 and CA3 and granule cell dispersion, HS is characterized by a chronic and fibrillary gliosis in CA1 and radial gliosis in the dentate gyrus, where the length of GFAP^+^ fibres is directly correlated with the degree of cell dispersion in the dentate gyrus (Fahrner et al., 2007). Furthermore, in HS, together with increased conventional GFAP expression, a novel GFAP isoform has been identified in small multinucleate CA1 and CA4 astrocytes, GFAP-γ, which is speculated to regulate astrocyte size and motility (Martinian et al., 2009). Whether HS is a primary cause of epilepsy or the result of repeated epileptic seizures is still controversial. Even if the prevailing view tends to consider HS as a secondary consequence of epilepsy, experimental data on surgical samples and autoptic tissues suggest that HS aetiology is multifactorial. Febrile seizures, genetic susceptibility, alterations of hippocampal development, head injuries, infections and inflammatory and neurodevelopmental factors have indeed been identified as predisposing elements to HS development (Sendrowski and Sobaniec, 2013, Thom, 2014, Walker, 2015).

### 3.2 Kir channels and K^+^ homeostasis

It is well known that astrocytes are key players in the regulation of extracellular K^+^ ([K^+^]_o_), which can transiently accumulate during prolonged neuronal activity and cause neuronal depolarization and hyper-excitability if uncompensated (Heinemann and Lux, 1977). [K^+^]_o_ homeostatic control is performed by K^+^ uptake and by spatial K^+^ buffering: while the former is mediated by glial and neuronal Na,K-ATPase or Na-K-Cl cotransporters, the latter is driven by the difference between the glial syncytium negative membrane potential and the local K^+^ equilibrium potential. This results in redistribution of K^+^ from sites of high neuronal activity to sites of lower [K^+^]_o_ through gap junction (GJ)-connected astrocytic networks (Orkand et al., 1966, Walz, 2000). This peculiar astrocytic property is mainly mediated by Kir4.1 K^+^ channels, which are weakly-rectifying channels possessing a high open probability at rest and showing conductance increase at high [K^+^]_o_ (Butt and Kalsi, 2006). Considering their role in K^+^ homeostasis and since increased levels of [K^+^]_o_ have been associated to the pathophysiology of epilepsy, Kir channels have been investigated in experimental (see (Chever et al., 2010, Djukic et al., 2007)) and human epilepsy. By using K^+^-sensitive microelectrodes, measurements of stimulus-induced [K^+^]_o_ increases have been performed in human sclerotic and non-sclerotic hippocampal tissues (HS and non-HS) in presence of Ba^2+^, a blocker of Kir channels (Kivi et al., 2000). These recordings highlighted an impaired K^+^ buffering in HS slices: indeed, they showed that Ba^2+^ was able to induce [K^+^]_o_ accumulation in hippocampal slices from lesion-associated TLE patients with no histopathological hippocampal alterations; on the contrary, it failed to do so in hippocampi from drug-resistant TLE patients showing strong neuronal loss and gliosis (Gabriel et al., 1998, Heinemann et al., 2000, Jauch et al., 2002, Kivi et al., 2000). Furthermore, patch-clamp recordings and single cell RT-PCR performed on human sclerotic hippocampus revealed a significant decrease (Hinterkeuser et al., 2000, Schröder et al., 2000) or a complete loss (Bordey and Sontheimer, 1998) of Kir4.1 currents; this has also been recently supported by Western blot and immunohistochemistry analysis showing a decrease of Kir4.1 protein levels in HS tissues compared to non-HS TLE patients or sudden-death controls (Das et al., 2012, Heuser et al., 2012). Thus, in HS TLE, the reduced expression and functionality of Kir4.1 channels determine an impaired K^+^ buffering and enhance seizure susceptibility, even if it is still controversial whether this reduction is a cause or a consequence of TLE.

Interestingly, several variations in human Kir4.1 gene (KCNJ10) have been identified and associated to different seizure phenotypes. Indeed, Buono and colleagues found that patients with refractory MTLE, childhood absence and juvenile myoclonic and idiopathic generalized epilepsy carried a missense mutation in the C-terminal domain of KCNJ10. Such domain is involved in ionic conductance, channel subunit dimerization and anchoring to the plasma membrane (Buono et al., 2004). Furthermore, other single nucleotide variations in non-coding regulatory KCNJ10 sequences have been found in TLE patients with febrile seizures (Heuser et al., 2010). Moreover, patients with EAST/SeSAME syndrome, an autosomal recessive multiorgan disorder characterized by seizures, ataxia, sensorineural deafness, mental retardation and electrolyte imbalance, presented loss-of-function mutations in highly conserved amino acid sequences of Kir4.1 gene, which causes impairment in channel conductance and Ba^2+^-sensitivity (Bockenhauer et al., 2009, Reichold et al., 2010, Scholl et al., 2009, Williams et al., 2010). Another cohort of patients displayed instead a gain-of-function mutation in KCNJ10 affecting the N-terminus and the first transmembrane region of the channel: this variation caused an increase in Kir4.1 surface expression or conductance and patients developed seizures in association with autism spectrum disorders and impaired cognitive abilities (Sicca et al., 2011).

### 3.3 Aquaporin-4 (AQP4)

Ion homeostasis in the brain depends not only on proper functioning of ion channels, but also on water transport. The influx of water between the blood and the brain parenchyma is tightly regulated by blood vessels. Glial ensheathment of blood vessels also contributes to water influx. The movement of water across cellular compartments is mediated by aquaporins (AQPs), transmembrane proteins which act as water channels in different cells and tissues. In the brain, the predominant form is AQP4, which is localized, as Kir4.1 channels, in astrocytic perivascular endfeet and perisynaptic processes. AQP4 participates in the control of extracellular fluid osmolarity and extracellular space volume by regulating water flow and K^+^ buffering (Nagelhus et al., 2004). It appears that water movement is altered in the hippocampus of HS TLE patients, as it has been shown that it presents an increased T2 signal density on magnetic resonance imaging and higher apparent diffusion coefficients, indicating water accumulation (Bronen et al., 1991, Hugg et al., 1999, Wieshmann et al., 1999). A potential impairment of AQP4 function has been proposed in TLE patient hippocampus: RT-PCR, immunohistochemistry and gene chip analysis have revealed an increased expression of AQP4 in HS tissues; this was accompanied by reduced levels of dystrophin, a protein involved in anchoring AQP4 to the membrane in perivascular endfeet (Lee et al., 2004). These results thus suggested an altered trafficking and distribution of AQP4 on the membranes. Quantitative electron microscopy later confirmed this hypothesis: the levels of AQP4 are indeed increased in MTLE compared to non-MTLE hippocampi. However, AQP4 density along the perivascular membrane domain of astrocytes was reduced by half in MTLE CA1 region, while no difference was found in AQP4 density on astrocyte membranes facing neuropil (Eid et al., 2005). These changes were secondary to altered perivascular dystrophin expression in sclerotic areas (Eid et al., 2005, Lee et al., 2004), which caused the loss of perivascular AQP4 and resulted in an impaired water flux through astrocytes. Given that in presence of high neuronal activity K^+^ and water are taken up by the astrocyte membrane facing the neuropil, transported through astrocytic syncytium and siphoned into blood or CSF through perivascular endfeet membrane (Paulson and Newman, 1987), this altered flow of water affected extracellular K^+^ buffering and contributed to epileptogenicity. Similar changes in AQP4 distribution have also been found in cortical samples of epileptic focal cortical dysplasia type IIb patients. AQP4 was indeed found to be enriched in the neuropil and around dysplastic neurons and reduced at the perivascular membranes due to disrupted dystrophin complex (Medici et al., 2011). The involvement of AQP4 in human epileptogenesis has been further confirmed by a genetic study which identified several polymorphisms of AQP4 gene associated to MTLE in combination with KCNJ10 single nucleotide polymorphisms (Heuser et al., 2010). These data support that alterations in water and K^+^ transport contribute to the aetiology of epilepsy.

### 3.4 Connexin43 and gap junctions

The role of GJ-connected astrocytic networks in the pathophysiology of epilepsy is still controversial, since they can act both as antiepileptic, by clearing and redistributing extracellular K^+^, glutamate and GABA (Orkand et al., 1966, Walz, 2000), and proepileptic, by fueling neurons with glucose and its metabolites in an activity-dependent manner (Rouach et al., 2008). Several studies have analysed changes in connexin (Cx) expression and coupling in human epileptic tissues: Naus and colleagues first reported increased Cx43 mRNA levels in the temporal cortex of epileptic patients (Naus et al., 1991). This change was confirmed by other groups both at the mRNA and protein levels (Aronica et al., 2001, Collignon et al., 2006, Fonseca et al., 2002). However, unchanged levels of Cx43 have also been reported in hippocampal tissues from patients presenting a complex partial seizure disorder in the medial temporal cortex and hippocampus (Elisevich et al., 1997). Whether expression and function of Cx30, the other astrocytic gap junction forming subunit, are altered in epilepsy remains unclear. Studies in rodents indeed report different results, which may be related to the diversity of the epilepsy models (Akbarpour et al., 2012, Condorelli et al., 2002, Söhl et al., 2000), and no data on human epileptic brain are yet available. Cx expression does not necessarily reflect active and functional coupling between astrocytes, since post-translational modifications can alter GJ properties, such as conductance, open probability and trafficking. Few functional data on GJ are available in human tissue. An early study performed *in vitro* on astrocytic cultures from tissues of MTLE patients reported an enhanced cell coupling by using the fluorescence recovery after photobleaching technique (Lee et al., 1995). However, recent work showed *ex vivo* a complete lack of glial GJ coupling in sclerotic hippocampal tissues from MTLE-HS patients, and a reduced coupling during the epileptogenic phase in the kainate mouse model of TLE (Bedner et al., 2015). It is unclear whether cultured astrocytes maintain the functional properties they display in the diseased tissue. Alternatively, gap junction coupling may be differentially regulated over the course of epilepsy, as increased astroglial coupling was found following status epilepticus in the kainate mouse (Takahashi et al., 2010).

### 3.5 Glutamate and GABA homeostasis

Neuronal activity leads to the release of excitatory and inhibitory neurotransmitters (mainly glutamate and GABA) in the extracellular space. To ensure appropriate synaptic responses and the maintenance of excitation-inhibition balance and to avoid neuronal loss and seizures, proper glutamate and GABA removal from the synaptic cleft becomes essential. Astrocytes play a crucial role in this phenomenon through the glutamine-glutamate-GABA cycle (Eid et al., 2012): extracellular glutamate and GABA released vesicularly are taken up by astrocytes via GLAST and GLT-1 glutamate transporters and GAT3 GABA transporter, respectively. Glutamine synthetase (GS) then directly converts glutamate into glutamine, while GABA enters the TCA cycle as succinate and is subsequently converted into alpha-ketoglutarate, glutamate, and glutamine. Specialized N-transporter proteins then extrude glutamine from astrocytes into the extracellular space, while glutamatergic and GABAergic neurons import glutamine through system A transporters, and convert it to glutamate and GABA, respectively. Using *in vivo* microdialysis, it has been shown that MTLE patients present five-fold higher extracellular glutamate levels in the epileptic sclerotic hippocampus compared to non-HS and non-epileptic hippocampal formation, despite neuronal loss and a two-fold increase in glial density (Cavus et al., 2005, Cavus et al., 2008). This suggested that an impaired neurotransmitter homeostasis could participate in the pathophysiology of epilepsy (Coulter and Eid, 2012). Indeed, alterations of glutamate and GABA transporters as well as GS have been identified in epileptic patients.

Studies on glutamate transporters in MTLE have reported contradictory results. An initial study found unchanged GLAST or GLT-1 expression through *in situ* hybridization and Western blot analysis in TLE hippocampus and cortex compared to post-mortem controls (Tessler et al., 1999). Similar results have also been reported in two other more recent studies (Bjørnsen et al., 2007, Eid et al., 2004). Conversely, a general decrease in GLT-1 and GLAST expression levels was found in HS TLE patients compared to non-HS TLE samples as assessed by immunocytochemical analysis (Mathern et al., 1999, Proper et al., 2002). This suggests that astrocytic glutamate uptake may play a crucial role in preventing epileptogenesis, even if the mechanism involved remains unknown.

Glutamate excess in TLE patients may be explained not only by a decreased expression in glutamate transporters, but also by impaired GS function. Indeed, it has been demonstrated that there is a reduction in GS expression and functionality in astrocytes of human sclerotic hippocampi (Eid et al., 2004, van der Hel et al., 2005): this results in slower rates of glutamate-glutamine cycling, accumulation of glutamate in astrocytic cytoplasm and decreased synaptic glutamate clearance (Petroff et al., 2002). On the contrary, subiculum astrocytes still express GS, but the protein is almost absent in most of the distal astrocytic processes, compared to non-epileptic controls (Eid et al., 2004). Furthermore, genetic mutations in GS gene (GLUL) have been found in epileptic patients: two GLUL congenital homozygous mutations occurring at GS active sites have been identified in two unrelated newborns, one of them displaying severe brain malformations, almost no EEG activity except short theta bursts and generalized seizures (Haber et al., 2006, Häberle et al., 2005). Noteworthy, a third mutation has recently been found in a child affected by epileptic encephalopathy and psychomotor retardation (Häberle et al., 2011).

A deficiency in GABA and GABA-mediated inhibition is thought to contribute to neuronal hyperexcitability in TLE. An *in vivo* microdialysis study has shown that the epileptogenic hippocampus of TLE patients presented lower extracellular GABA levels and higher glutamate concentrations just before the onset of seizures compared to the non-epileptogenic hippocampus of the same patient (During and Spencer, 1993). Interestingly, it has been demonstrated that this was due to an increased expression of the astrocytic GABA transporter GAT3, which is normally only weakly expressed in human hippocampal astrocytes (Lee et al., 2006): indeed, GAT3 was more prominently expressed in cells resembling protoplasmic astrocytes, located in dentate gyrus and hilus of sclerotic hippocampal formations. This increased GAT3 expression may thus explain GABA reduced extracellular levels during seizures, via an increased uptake by astrocytes (During and Spencer, 1993). Differently from TLE, absence epilepsy is characterized by an increased tonic GABA_A_ receptor-mediated inhibition in thalamocortical neurons, which is necessary and sufficient for the generation of non-convulsive seizures, typical of this form of epilepsy. This alteration of GABA levels is caused by a dysfunction of GABA transporter GAT-1, which is exclusively located in astrocytes in the thalamus of both humans and rodents (Crunelli et al., 2011, Pirttimaki et al., 2013).

Altogether these results indicate that an impairment of glutamate and GABA extracellular levels, due to altered expression of their transporters and reduced expression and functionality of GS, can play a major role in the pathophysiology of epilepsy.

### 3.6 Membrane channels

Astrocytes of the sclerotic epileptic hippocampus exhibit unusual properties compared to astrocytes of other non-epileptic brain regions, due to alterations of plasma membrane channels and receptors. For instance, it has been shown that astrocytes in the hippocampus of HS-TLE patients present higher expression of metabotropic glutamatergic receptors mGluR2/3, mGluR4 and mGluR8 (Tang and Lee, 2001).

Several studies using patch-clamp have also demonstrated an altered expression of voltage-gated channels in astrocytes of the epileptic hippocampus. A dramatic increase in Na^+^ current density was indeed found in cultures of MTLE astrocytes, which displayed a depolarized membrane potential and action potential-like responses when stimulated with current injections (O’Connor et al., 1998). Similar results were also reported in human acute hippocampal slices from MTLE patients (Bordey and Sontheimer, 1998, Bordey and Spencer, 2004): in these tissues astrocytes presented complex, arborized and highly branched processes intensively stained for GFAP, and expressed high levels of TTX-sensitive Na^+^ channels, allowing generation of slow action potentials. A significant up-regulation of the α_1C_ subunit of voltage-gated Ca^2+^ channels was also observed as assessed by immunohistochemistry on sclerotic TLE hippocampi (Djamshidian et al., 2002); this altered the properties of L-type Ca^2+^ currents, suggesting an increased astrocytic Ca^2+^ uptake.

All in all, investigations of brain tissue samples from epileptic patients have revealed alterations in expression, localization and functionality of several astrocytic proteins, such as Kir4.1 channels, AQP4, Cx43, glutamate and GABA transporters. In addition, an enhanced expression of voltage-gated channels, expressed at low levels by astrocytes in the healthy brain, has also been observed in astrocytes from epileptic brain specimens. Accordingly, dysfunctional astrocytes can play a crucial role in the process of epileptogenesis and can thus be considered as alternative targets to develop new antiepileptic drugs.

## 4 Brain tumours

Gliomas, representing the majority of primary brain tumours, mainly originate from glial cells. They are one of the most aggressive neoplasias, since they carry a poor prognosis despite aggressive therapies due to their ability to infiltrate the brain and grow. They are classified according to the morphological properties of the tumour-forming cells (Louis et al., 2007). In particular, astrocyte-resembling cells are responsible for the development of astrocytomas, which represent the most common subtype of brain gliomas (Furnari et al., 2007). Several studies have identified astrocytic morphological and functional changes in astrocytoma, mainly affecting extracellular glutamate levels and membrane channel functions (Fig. 2c).

### 4.1 Structural and morphological changes of astrocytes

Glioma cells share many characteristics with non-tumoural astrocytes, but also present several structural and morphological differences. In tissue of glioma patients, an overexpression of GFAP has been identified as being positively correlated with glioma size, but not with the degree of malignancy (Herpers et al., 1986, Lee et al., 2011a). For this reason, GFAP is used as a reliable immunohistochemical marker to stain surgically resected brain tumours in order to verify their astrocytic origin (Vinters et al., 1998). A strong GFAP immunoreactivity is present also in reactive astrocytes within or surrounding non-glial tumours (Raore et al., 2011). In grade I to IV astrocytomas, GFAP and vimentin are co-expressed but in different cellular compartments: indeed, while vimentin is located closer to cell nucleus, GFAP is preferentially detected in cellular processes (Bordey and Sontheimer, 1998, Cosgrove et al., 1989, Herpers et al., 1986). Besides GFAP, changes in other types of intermediate filament structural proteins have been observed: astrocytoma cells express the vimentin binding, 300-kDa intermediate filament associated protein (IFAP–300 kDa), which is normally present only in radial glia and immature astrocytes and absent in normal adult brain (Yang et al., 1993, Yang et al., 1994); furthermore, in astrocytomas, the proportion of keratin-containing cells, which are normally detected in the neuroectoderm, is directly linked to the degree of tumour malignancy, thus supporting a dedifferentiation of tumoural cells (Yang et al., 1994).

The modified expression of cytoskeletal elements may be responsible of the altered structure of astrocytic glioma cells: indeed, compared to non-tumoural astrocytes, which present a stellate shape with 2–3 major and many smaller processes, low-grade pilocytic astrocytoma cells display only 2–3 thick processes (Bordey and Sontheimer, 1998). Furthermore, astrocytic cells in low-grade gliomas display minimal to moderate nuclear atypia, scant cytoplasm and a high nucleus-to-cytoplasm ratio (Burel-Vandenbos et al., 2011).

### 4.2 Extracellular glutamate

Extracellular glutamate concentration is elevated in tumoural and peritumoural regions, especially close to tumours containing necrotic areas in high-grade astrocytoma patients (Roslin et al., 2003). Similar results have also been observed in oligodendrogliomas, which present high levels of glutamate and glutamine in the peritumoural area, as assessed by magnetic resonance spectroscopy (Rijpkema et al., 2003). This altered glutamate homeostasis explains why during the time course of the disease, 60–80% of glioma patients experience seizures (Kurzwelly et al., 2010, Lynam et al., 2007), which originate close to the tumour mass (Pallud et al., 2013, Patt et al., 2000). Various studies aiming at identifying the source of peritumoural glutamate reported an impaired expression of glutamate transporters on glioma cells: brain tissues from glioblastoma patients indeed display a strong reduction in GLT-1 levels, while GLAST is normally expressed but is thought to be mislocalized in cell nuclei rather than at the plasma membrane (Lynam et al., 2007, Savaskan et al., 2008, Ye et al., 1999). Decreased GLT-1 levels have also been observed in high-grade compared to low-grade astrocytomas and normal brains (de Groot et al., 2005). Furthermore these changes are accompanied by an altered expression of the cysteine-glutamate system (xc system), a Na^+^-independent exchanger that controls the intracellular glutathione levels by importing one molecule of extracellular cysteine (required in glutathione synthesis) per released glutamate. Glioma cell lines originated from tumours and brain specimens from glioblastoma patients express the xc system at significantly higher levels compared to human tissue samples without malignant transformation (Savaskan et al., 2008, Ye et al., 1999). Furthermore, a recent study has shown that 50% of patient-derived gliomas have elevated expression of SLC7A11, the catalytic subunit of the xc system responsible for xc-mediated glutamate release (Robert et al., 2015). Interestingly, when these glioma cells implanted intracranially in mice propagated *in vivo* as flank tumour xenolines, they caused seizures, tumour-associated excitotoxicity and shortened survival. Altogether these results thus suggest that high levels of this system contribute to the release of cytotoxic glutamate levels, which promote seizures and act as an autocrine/paracrine signal sustaining tumour growth and invasion (Lyons et al., 2007).

### 4.3 Gap-junctions and membrane ion channels

Normal growth and metabolism of cells depend not only on their organelles and subcellular structures, but also on cell-to-cell communication, in which GJs play a fundamental role. Impairment of GJ-mediated intercellular communication may indeed result in aberrant growth and tumour development (Omori and Yamasaki, 1998). Noteworthy, low-grade gliomas present a strong Cx43 immunoreactivity, particularly in reactive astrocytes of the peritumoural area; on the contrary, in surgical specimen from high-grade astrocytoma patients Cx43 levels are reduced both on membranes and in the cytoplasm. In addition, only the non-phosphorylated isoform of Cx43 was detected (Aronica et al., 2001). Similar results have also been observed in primary astrocytic cultures from glioblastoma multiform patients (Soroceanu et al., 2001), where reduced Cx43 expression and GJ-mediated coupling were found. Interestingly, the decrease in Cx43 expression is proportional to tumour grade and proliferative capacity (Pu et al., 2004), and is not due to a reduced genetic transcription: grade III and IV gliomas indeed present elevated Cx43 mRNA but low proteins levels (Caltabiano et al., 2010), suggesting an alteration in post-transcriptional mechanisms in glioma astrocytic cells.

Besides GJ channels, astrocytic tumoural cells present changes in the expression of Na^+^ and K^+^ channels: low-grade pilocytic astrocytoma cells indeed display almost exclusively delayed rectifying K^+^ currents, while no transient A-type and inwardly rectifying K^+^ channels were detected. Alteration in K^+^ channel-dependent cell volume regulation resulted in a depolarized membrane potential and a round swollen cell body. Furthermore, these cells have increased TTX-sensitive Na^+^ currents, which enable them to generate spike-like events after current injections (Bordey and Sontheimer, 1998). Alterations in Na^+^ channel expression have also been found in high-grade gliomas. Pleomorphic GFAP^+^ cells and hypertrophic reactive astrocytes adjacent to multiform glioblastoma showed strong Nav1.5 expression in cell bodies and processes, compared to astrocytes in normal white matter (Black et al., 2010).

Malignant astrocytic gliomas, characterized by uncontrolled cellular proliferation and diffuse infiltration, show an intense resistance to apoptosis, which contributes to the ineffectiveness of traditional therapeutic approaches (such as surgical resection, radiotherapy and chemotherapy). Astrocytes in these cancers display morphological changes and increased GFAP expression, two phenotypes that have been described in activated astrocytes after a CNS injury. Moreover, GJ coupling is reduced: this favors malignant transformation via a reduction of inhibitory signals controlling cell division and proliferation received from neighbouring cells. Glioma astrocytes also present impaired extracellular glutamate regulation and aberrant expression of voltage-gated channels, contributing to aberrant neuronal and astrocytic activity. In order to consider these dysregulated astrocytic pathways as potential therapeutical targets, it is of crucial importance to understand how glioma cells modify during tumour progression and interact with neighbouring normal and cancerous cells in the tumour microenvironment.

## 5 Alzheimer’s disease

Alzheimer’s disease (AD) is the most common type of dementia in elderly, accounting for 60–80% of dementia patients (Wortmann, 2012). It is characterized by a subtle decline in episodic memory, appearing as a deficit in recalling the recent past, followed by a more global decline of cognitive abilities, such as loss of long-term memories, language, attention and personality changes (Querfurth and LaFerla, 2010). AD is identified by two histopathological hallmarks, extracellular deposits (plaques) of amyloid-beta (Aβ) protein and intracellular neuronal tangles formed by abnormally phosphorylated tau protein (Querfurth and LaFerla, 2010). The distribution pattern of neurofibrillary tangles and neuronal alterations are generally used to define the stage of AD progression (Braak and Braak, 1991, Braak and Braak, 1995). In stages I and II, neuronal alterations are confined to the transentorhinal region, while both the entorhinal/transentorhinal areas and hippocampus are involved in stages III and IV. The last stages of AD progression (V and VI) are instead marked by devastating neocortical distruction and represent the fully developed AD (Braak and Braak, 1995). Despite the global economic burden of this disease, effective treatments are still lacking and the causes of the disease remain elusive. The pathological potential of astrocytes in AD has been initially suggested in 1910 by Alois Alzheimer, who found glial cells in closed association with damaged neurons and abundantly populating senile plaques (Verkhratsky et al., 2010). This has been subsequently confirmed by studies on human tissues and AD animal models showing astrocytic hypertrophy, particularly in astrocytes associated with senile plaques (Nagele et al., 2004), as well as glial changes often preceding plaque and tangle formation (Rodríguez-Arellano et al., 2016) (Fig. 2d).

### 5.1 Astrogliosis in AD

Reactive astrogliosis is a well-known hallmark of AD, even if its role has not been clearly understood yet. It is identified by an increased expression of GFAP and hypertrophy of astrocytes in the vicinity of amyloid plaques. Post-mortem tissues from AD patients indeed display increased GFAP levels in temporal (Griffin et al., 1989, Simpson et al., 2010), occipital, parietal and frontal lobes (Kashon et al., 2004). Moreover, in the cerebrospinal fluid of AD patients, higher levels of GFAP concentrations have been measured compared to age-matched controls (Jesse et al., 2009). Interestingly, some degree of correlation has been found between GFAP expression and AD progression, with higher GFAP levels at increasing Braak groups (Simpson et al., 2010) or duration of clinical illness (Serrano-Pozo et al., 2013). However, this correlation remains uncertain, since another work showed no difference in GFAP expression between demented and non-demented brains within the same Braak stage (Wharton et al., 2009). During AD progression, 8 of the 10 different GFAP isoforms described (Hol and Pekny, 2015) are upregulated. For instance, reactive astrocytes in dentate gyrus subgranular zone, hilus and CA4 area of AD patients display a prominent expression of GFAPδ, but only CA1, CA3 and subiculum astrocytes surrounding plaques showed GFAPδ upregulation with increasing AD stage (Kamphuis et al., 2014). Additionally, the number of human-specific astrocyte subtypes expressing the frame-shifted GFAP variant, GFAP^+1^, is increased with AD progression, but only few of these GFAP^+1^-expressing cells has been identified as associated to plaques, with processes protruding through them (Kamphuis et al., 2014, Middeldorp et al., 2009). Astrogliosis and GFAP upregulation are also accompanied by dysregulation in the expression of other astrocytic cytoskeleton proteins. For instance, in the lateral temporal cortex of advanced AD stages, there is a significant decrease in transcripts encoding members of the myosin and kinesin family and other cytoskeletal proteins, such as actin β, dynein and integrin α. Moreover, transcripts encoding tight junction proteins and adherens junctions are also reduced during AD progression (Simpson et al., 2011). The effect of the altered expression of these genes in astrocytes still remains unclear, but it may affect various intracellular signalling pathways.

### 5.2 Interactions between astrocytes and Aβ

In AD brains at early stages of the pathology, activated reactive astrocytes are predominant in the molecular layer of the cerebral cortex and close to amyloid plaques in pyramidal cell layers (Wisniewski and Wegiel, 1991). In several brain regions such as cortex, hippocampus and cerebellum, proliferating processes of hypertrophic astrocytes nearest to amyloid deposits contact and surround the plaques; they penetrate more into non-cored primitive plaques, compared to classic compact cored amyloid deposits, thus merging with them and contributing to their fragmentation, dispersion and the observed variety of plaque morphology (Kato et al., 1998, Wisniewski and Wegiel, 1991). Interestingly, in the visual cortex of AD brains with severe pathology, GFAP-immunoreactive astrocytes and plaques are arranged in a specific laminar distribution: indeed gliosis is preferentially localized in laminae II, III, IVa and IVc, the latter presenting a discrete plaque-associated glyotic horizontal band at the lower edge (Beach and McGeer, 1988). Furthermore, a more recent study has shown that reactive astrocytes together with microglial cells form specific 3D reactive glial nets around plaques in a plaque-specific way: at Aβ dense-core plaques, astrocytic processes are intermingled with microglial cell bodies which envelop the core Aβ structure; while at fibrillary plaques, a higher number of glial cells are recruited to reactive glial net formation and both microglial and astrocytic processes invade the plaque area and interact with Aβ protein (Bouvier et al., 2016).

The stimulus capable of inducing astrocyte reactivity in AD brains is still under debate; however, studies using aggregated Aβ protein and the intact core of Aβ plaques isolated from AD brain tissue have shown that Aβ can trigger activation of astrocytes *in vitro*, causing GFAP up-regulation and morphological changes (DeWitt et al., 1998). Furthermore, amyloid plaques colocalize with reactive astrocytes in the absence of dystrophic neurites in the hippocampus of mild AD brains, while decreasing over the course of the pathology (Pike et al., 1995). This suggests that plaque-associated astrocytosis can act as a contributory event in AD pathology and that once activated, astrocytes can participate in Aβ metabolism. Aβ42 indeed accumulates in the cytoplasm and processes of reactive astrocytes in the molecular layer completely devoid of amyloid plaques in AD brains. Aβ42 also builds up in astrocytes associated with plaques in pyramidal cell layers of the entorhinal, parietal, occipital and temporal cortex (Akiyama et al., 1999, Funato et al., 1998, Kurt et al., 1999, Nagele et al., 2003, Thal et al., 1999, Thal et al., 2000). In these cells, non-fibrillar Aβ42 localizes to small lysosomal lipofuscin-like granules, organized in clusters and mainly located in the perinuclear cytoplasm (Funato et al., 1998, Nagele et al., 2003, Thal et al., 1999, Yamaguchi et al., 1998). Moreover, it has recently been reported that more than 90% of activated astrocytes in AD brain frontal cortex can accumulate a newly described class of amyloid structures formed by Aβ, the annular protofibrils. These fibrils are absent in amyloid plaques and in the brain of age-matched controls, and can induce reactive oxygen species generation and inactivation of GS in the cell (Lasagna-Reeves and Kayed, 2011).

The accumulation of Aβ42 in astrocytes is directly linked to the severity of local AD pathology. Aβ42^+^ material in astrocytes is indeed proportional to the amount of Aβ42 contained in surrounding neurons and to the local density of amyloid plaques within the pyramidal cell layer of the entorhinal cortex, while the amount of Aβ42 in astrocytes of the molecular layer devoid of plaques well correlates with the severity of the pathology in the sub-adjacent cortical laminae (Nagele et al., 2003). Interestingly, astrocyte-accumulated Aβ42 is not produced by astrocytes, but has a neuronal origin, deriving from the internalization of degenerating synapses and dendrites by phagocytosis: astrocytic Aβ42 colocalizes with choline acetyltransferase and α7 nicotinic acetylcholine receptors, which are neuron-specific proteins accumulating in astrocytes as a consequence of their debris-clearing activity (Nagele et al., 2004). Furthermore, with the progression of the disease, Aβ42-burdened astrocytes can undergo lysis and form small spherical GFAP^+^ amyloid plaques, first appearing in the subpial portion of the cortical molecular layer close to astrocytes containing large Aβ42 deposits (Nagele et al., 2003).

These results indicate that not only neurons, but also astrocytes are capable of giving rise to amyloid plaques and causing morphological modifications within these plaques; this ability can thus account, at least in part, for the variety of plaque morphology identified in AD brains. Blocking the initial accumulation of Aβ42 in neurons represents the main early target to control AD pathology. However, in view of the ability of astrocytes to interact with plaques and participate in Aβ metabolism, limiting their recruitment could also contribute to limiting or delaying AD progression.

### 5.3 Glutamate and GABA homeostasis

There are several indications that astrocytic glutamatergic function is impaired in AD. GLT-1 immunoreactivity is reduced in the frontal cortex of AD patients (Li et al., 1997, Tian et al., 2010) and this reduction is inversely correlated with amyloid precursor protein mRNA levels, while no change has been observed for GLAST expression. GLT-1 reduced expression is also accompanied by decreased glutamatergic transport activity and increased mRNA levels, thus indicating an impairment at post-transcriptional level (Li et al., 1997). Similar results have also been obtained in the hippocampus and gyrus frontalis of AD patient brains. A marked impairment in GLT-1 and GLAST expression at both gene and protein levels occur already at early stages of the disease, particularly in the vicinity of amyloid plaques (Jacob et al., 2007). Furthermore, in AD lateral temporal cortex, GLT-1 expression tends to decrease with higher Braak group. This correlation is however still under debate, since contradictory results have been obtained due to high variability of glutamate transporter expression between AD individuals (Beckstrøm et al., 1999). Together with reduced expression, protein splice variants can also account for changes in the functionality of glutamate transporters. Interestingly, disease- and pathology-specific changes in GLT-1 splice variant expression occur in autoptic AD brains, which can account for the reduced astrocytic glutamate uptake efficiency in AD. In particular, a reduction in the functional splice variants b, which is able to uptake glutamate, has been identified in several brain regions, together with a significant increase of exon-skipping variants, characterized by reduced transport capacity (Scott et al., 2011). Altogether, these results suggest an impairment of astrocytic glutamate clearance capability in AD, which may lead to neurotoxic effect and contribute to the increased prevalence of seizures in AD patients (Scarmeas et al., 2009).

Furthermore, the levels of GS are also altered in AD brain: temporal neocortex astrocytes from AD brain samples display reduced GS expression due to impairment of post-translational modifications, which renders the protein highly sensitive to oxidative lesioning (Le Prince et al., 1995). In contrast, total GS levels in the CSF of AD patients remain unchanged (Timmer et al., 2015).

GABAergic dysfunction also plays a role in AD, and several recent studies point to abnormalities of GABA homeostasis in reactive AD astrocytes. An accumulation of GABA together with an increase in GAD67 and GAT3 have been found in GFAP^+^ astrocytes of dentate gyrus molecular layer and inferior temporal cortex from post-mortem AD patients, but not in age-related healthy subjects (Mitew et al., 2013, Wu et al., 2014). This suggests an increased GABA production in astrocytes. Consistent with this hypothesis, astrocytic monoamine oxidase-B, the enzyme responsible for GABA production, is up-regulated in post-mortem brains of AD individuals. This has also been shown in AD mouse models, where accumulating GABA is abnormally released from reactive astrocytes through bestrophin 1 channels, which impairs learning and memory (Jo et al., 2014) The high astrocytic GABA level identified in human brains with high Aβ load may thus be used as a novel biomarker and diagnostic tool for AD. Furthermore, since it has been shown that GABA transporters can reverse their transport direction in presence of excessive intracellular GABA (Lee et al., 2011b, Richerson and Wu, 2003), GAT3 may serve as a new drug target.

### 5.4 Astrocyte-specific protein changes

Not only are glutamate and GABA homeostasis altered in AD, but also other astrocytic-specific functions, such as GJ communication and K^+^ buffering. In 1996 Nagy and colleagues reported for the first time an increased Cx43 expression in temporal cortical areas containing several amyloid plaques in post-mortem AD human brains. Furthermore, they also demonstrated using electron microscopy that the enhanced Cx43 immunoreactivity was restricted to astrocytic GJs throughout the tissue, both within and outside plaque-containing regions (Nagy et al., 1996). This observation was later confirmed, as a similar enrichment of Cx43 puncta occurred in reactive astrocytic processes infiltrating amyloid plaques, together with a milder increase of Cx30 (Koulakoff et al., 2012). AD brain tissues also display altered Kir4.1 and AQP4 expression (Wilcock et al., 2009): a strong decrease in Kir4.1 and AQP4 mRNA levels occur in the temporal cortex of brain samples from AD patients with moderate and severe pathology. Furthermore AQP4 is also mislocalized, and presents a diffuse staining pattern with poorly distinguishable blood vessels in severe pathology cases (Wilcock et al., 2009). A significant increase in AQP1, normally expressed in the choroid plexus, has been identified in astrocytes of the frontal cortex and temporal lobes in sporadic and familial AD cases, where AQP1^+^ astrocytes were found close to Aβ42 or Aβ40 deposits (Hoshi et al., 2012, Pérez et al., 2007).

A deregulation of cellular Ca^2+^ levels has been proposed to contribute to the initial steps of AD progression, by causing a long-lasting overload in the cytoplasm and the endoplasmic reticulum, which triggers cell death. Although this signalling was considered compromised mainly in neurons, astrocytes are also affected as demonstrated in rodent AD models and AD human brain samples. Basal Ca^2+^ levels are indeed elevated in the astrocytic network and Ca^2+^ transients are more frequent, coordinated across long distances and independent from neuronal activity in the cortex of AD mice (Kuchibhotla et al., 2009). An enhanced Ca^2+^ response may be the result of an altered expression of astrocytic metabotropic glutamate receptor 5 (mGluR5). Indeed, an increased mGluR5 staining has been identified in hippocampal astrocytes of AD patients, in proximity of Aβ plaques (Lim et al., 2013). Furthermore, changes in S100β levels have been identified in AD patients: while S100β is strongly overexpressed in AD hippocampal and temporal lobe astrocytes closely associated with either diffuse or neuritic Aβ plaques, only moderate increases have been found in frontal lobe and pons and unchanged levels in occipital lobe and cerebellum (Griffin et al., 1989, Marshak et al., 1992, Van Eldik and Griffin, 1994). In the temporal lobe from AD human brain, S100β-overexpressing astrocytes are mainly associated with diffuse neuritic plaques, while diffuse non-neuritic and dense-core neuritic plaques have small numbers of associated S100β astrocytes. Astrocytes were observed in close proximity to dense-core non-neuritic plaques only rarely (Mrak et al., 1996). This indicates that activated astrocytes producing S100β are present already during the earliest stages of plaque formation, and decrease at the end-stage of plaque progression. Furthermore, a correlation has been found between neuritic plaque density and S100β levels, which are significantly increased in the brain of AD patients compared to age-matched controls (Mrak et al., 1996). The number of activated S100β-overexpressing astrocytes associated with single neuritic plaques and the degree of neuritic pathology in the same plaques are also correlated (Mrak et al., 1996, Sheng et al., 1994, Sheng et al., 1996). Remarkably, AD mice also display enhanced glutamatergic gliotransmission, as indicated by the increased frequency in resting conditions of the slow inward currents mediated by activation of NMDA receptors in neurons (Gómez-Gonzalo et al., 2017). Furthermore, astrocytic Ca^2+^ signals can be protective during the initial phase of AD, since the disruption of inositol triphosphate receptors 2 (IP3R2)-mediated Ca^2+^ signalling in astrocytes boosted the progression of Aβ plaque deposition and synaptic plasticity dysfunction at very early stages of the pathology (Gómez-Gonzalo et al., 2017).

During AD progression, astrocytes display a complex pattern of dysfunctions, concerning cytoskeleton, cell junctions, gap junction communication, intracellular signalling molecules and neurotransmitter homeostasis. These pathways are progressively affected with increasing Braak stages of the pathology, thus suggesting a continuous decline of astrocyte functions in AD. Further investigations are now required to clarify whether and which of these changes play an active role in AD development. This will be crucial in designing new therapies aimed at rescuing astrocytic physiological functions that may limit or even prevent AD-associated cognitive decline.

## 6 Major depressive disorder

Major depressive disorder (MDD), a chronic recurrent and debilitating mental illness, is characterized by depressed mood, loss of interest and pleasure, weight changes, sleep alterations, loss of energy, difficulties of concentration and thought of death and suicide (American Psychiatric Association, 2013). Numerous studies have revealed that MDD is a disorder with prominent pathological astrocytic alterations, which affect density, morphology, protein expression and membrane channel functions of astrocytes (Fig. 2e). However, astrocytic changes in MDD strongly differ from what is observed in other neurological and neurodegenerative disorders, such as epilepsy, inflammation and Alzheimer’s disease: while these diseases present reactive astrogliosis, glial scar formation and neuronal loss (Sofroniew, 2009, Sofroniew and Vinters, 2010), astrogliosis and prominent neuronal pathology is not present in MDD.

### 6.1 Astrocyte density and morphology

Many histopathological studies performed on post-mortem brain samples have unveiled prominent decreases in astrocyte number and packing density in MDD subjects compared to age-matched non-psychiatric controls (Cotter et al., 2002, Cotter et al., 2001, Gittins and Harrison, 2011, Ongür et al., 1998, Rajkowska et al., 1999). Several brain regions display a reduced astrocytic population, such as dorsolateral prefrontal (Cotter et al., 2002, Rajkowska et al., 1999), orbitofrontal (Rajkowska et al., 1999), subgenual (Ongür et al., 1998) and anterior cingulate cortex (Cotter et al., 2001) and amygdala (Altshuler et al., 2010, Bowley et al., 2002). However, an increase in glial cell density has also been reported in hippocampal regions and dentate gyrus of MDD patients (Stockmeier et al., 2004), while no change has been observed in the orbitofrontal cortex and in the supragenual region of the anterior cingulate cortex in late-life depressed patients (Khundakar et al., 2011a, Khundakar et al., 2011b) and in the hippocampus (Cobb et al., 2013). Changes in glial density may thus differentially affect specific brain regions. Interestingly, it has been shown that this alteration is age-dependent: in grey matter of dorsolateral prefrontal cortex of younger depressed patients (<50 years old), the density of GFAP^+^ astrocytes is significantly reduced compared to controls of similar age; in contrast, older subjects with late-onset depression presented increased astrocytic population in the same area (Miguel-Hidalgo et al., 2000), probably reflecting a compensation to neuronal loss observed in older MDD patients (Rajkowska et al., 2005).

In parallel to alterations of astrocyte packing density, the size of glial nuclei seems to be affected in MDD. Astrocytes with larger nuclei have been observed in the dorsolateral prefrontal cortex (Rajkowska et al., 1999) and in the grey and white matter of the anterior cingulate cortex: in these regions fibrous astrocytes had bigger cell bodies and more ramified processes in depressed subjects committing suicide compared to matched sudden-death controls (Chana et al., 2003, Torres-Platas et al., 2011). On the contrary, three other studies observed unaltered glial size in the prefrontal and orbitofrontal cortex and in the hippocampus (Cotter et al., 2002, Cotter et al., 2005, Stockmeier et al., 2004).

Studies on GFAP expression additionally revealed marked alterations in MDD subjects. Immunohistochemical analysis aimed at quantifying the area covered by GFAP^+^ cell bodies and processes has shown a predominant decrease of GFAP in grey matter of the prefrontal cortex in young depressed subjects compared to controls (Miguel-Hidalgo et al., 2000), in white matter of anterior cingulate cortex (Gittins and Harrison, 2011), in orbitofrontal cortex (Miguel-Hidalgo et al., 2010), CA1 and CA2 hippocampal regions (Müller et al., 2001), locus coeruleus (Chandley et al., 2013) and cerebellum (Fatemi et al., 2004). Moreover, GFAP decrease has also been confirmed at mRNA and protein levels (Chandley et al., 2013, Fatemi et al., 2004, Johnston-Wilson et al., 2000, Miguel-Hidalgo et al., 2000, Webster et al., 2005) and it is correlated with age and onset of depression: the levels of GFAP protein are indeed significantly lower in less than 60 years-old depressed patients compared to age-matched controls, with no change observed between older MDD subjects and their controls (Si et al., 2004). In contrast, an increase of GFAP occurred in dorsolateral prefrontal cortex in late-onset MDD patients (Davis et al., 2002, Miguel-Hidalgo et al., 2000).

### 6.2 Glutamate and GABA homeostasis

Recent neuroimaging and post-mortem studies on depressed subjects have revealed a dysfunction of astrocytic-mediated regulation of glutamate homeostasis. MDD subjects presented lower levels of glutamate, glutamine or combined glutamate/glutamine (Glx), as assessed by magnetic resonance spectroscopy, in several brain regions such as prefrontal areas (Hasler et al., 2007), frontal lobe (Yildiz-Yesiloglu and Ankerst, 2006), anterior cingulate cortex (Auer et al., 2000, Mirza et al., 2004, Pfleiderer et al., 2003) and amygdala (Michael et al., 2003), and in plasma (Altamura et al., 1995). This suggests an impaired glutamate/glutamine metabolism. These changes may however not appear homogenously in all brain areas and in all depressed patients, since two other studies reported glutamate increases in occipital and frontal cortex (Hashimoto et al., 2007, Sanacora et al., 2004), probably reflecting region-specific alterations which can also depend on patient age and chronicity of depression.

The impaired glutamate homeostasis observed in MDD patients may be related to the reduced astrocytic packing density detected from post-mortem studies on MDD brain samples. Furthermore, reduced expression of GLAST and GLT-1 occur in anterior cingulate (Choudary et al., 2005), dorsolateral prefrontal (Choudary et al., 2005, Klempan et al., 2009) and orbitofrontal cortex (Miguel-Hidalgo et al., 2010), and in locus coeruleus (Bernard et al., 2011, Chandley et al., 2013) and hippocampus (Medina et al., 2016) of subjects diagnosed with MDD. Finally, different cortical and subcortical regions of depressed suicide victims, such as prefrontal and premotor cortex and the amygdala, also display reduced levels of GS (Miguel-Hidalgo et al., 2010, Sequeira et al., 2009). Interestingly, it has been shown that the impairment in glutamate-related gene expression is specific to astrocyte in MDD, since it does not occur in neurons of MDD brains (Bernard et al., 2011), thus underlying the astrocytic basis of MDD pathology.

In MDD not only glutamate regulation is impaired, but also GABA homeostasis. Indeed, reduced GABA levels have been found in the dorsomedial/dorsal anterolateral prefrontal cortex (Hasler et al., 2007), and in the occipital cortex (Sanacora et al., 2004) of depressed but not remitted MDD patients (Schür et al., 2016). Furthermore, in the dorsolateral prefrontal cortex, GABA_A_ receptor subunits are up-regulated (Choudary et al., 2005, Sequeira et al., 2009). Interestingly, it has been shown that α1 and β3 subunit expression is selectively increased in suicide completers (Choudary et al., 2005).

### 6.3 Membrane channels and proteins

Several astrocytic-specific membrane channels have been demonstrated to be altered in MDD subjects. A decrease in gene and protein expression of Cx43 and Cx30 occurs in the dorsolateral prefrontal cortex of suicide completers (Ernst et al., 2011) and in the locus coeruleus of MDD patients (Bernard et al., 2011). Cx43 levels are also reduced in the orbitofrontal cortex (Miguel-Hidalgo et al., 2014) and hippocampus (Medina et al., 2016).

Astrocytes in MDD subjects display altered K^+^ and water homeostasis: Kir4.1 channels are down-regulated in the hippocampus of depressed patients (Medina et al., 2016); AQP4 immunoreactivity and mRNA levels decrease in the grey matter of orbitofrontal cortex, where the coverage of blood vessels by astrocytic endfeet is reduced (Rajkowska et al., 2013), in locus coeruleus and in the hippocampus of MDD patients, compared to psychiatrically-normal control subjects (Bernard et al., 2011, Medina et al., 2016).

Finally, another astrocytic marker, the Ca^2+^ binding protein S100β, which is predominantly expressed and secreted by grey matter astrocytes and involved in several Ca^2+^-dependent intracellular functions, is affected in MDD pathology. S100β mRNA levels are decreased in the prefrontal cortex and hippocampus of depressed suicide victims (Bernard et al., 2011, Klempan et al., 2009), and the number of S100β^+^ astrocytes is strongly reduced in CA1 hippocampal region of MDD patients (Gos et al., 2013). Furthermore, in agreement with MDD astrocytic pathology, S100β cerebrospinal fluid and serum levels are increased (Grabe et al., 2001, Rothermundt et al., 2001, Schroeter et al., 2002, Schroeter et al., 2008), probably reflecting leakage of S100β from astrocytic cytoplasm into extracellular compartments.

To summarize, key features of MDD include a reduction in astrocyte population and alterations in the expression of several astrocytic markers, such as GFAP, GJ proteins, AQP4, Kir4.1 channels, S100β and glutamate transporters. MDD patients do not present prominent astrogliosis, glial scar formation and neuronal degeneration, which are conversely observed in other brain disorders, such as tumours, AD, amyotrophic lateral sclerosis and Huntington’s disease. Astrocytes can thus be considered as novel targets for antidepressant medications. Nonetheless, the reciprocal communication between astrocytes and neurons in MDD remains still unclear; moreover, it would be interesting to determine whether alterations of astrocytic physiology and number are present only during episodes of depression or also during periods of remission.

## 7 Down syndrome

Down syndrome (DS) is the most common well-known chromosomal disorder, affecting one in every 700 babies in the United States (Parker et al., 2010). DS is caused by the trisomy of chromosome 21, and characterized by mental retardation, language impairment and other phenotypic abnormalities, such as slanting eyes, flat facial feature and hypotonia. Furthermore, in addition to developmental failure, DS is characterized by AD-like pathology, with neuritic Aβ plaques widely developing in the hippocampus and enthorinal cortex of almost all adults with DS and in some DS children (Hof et al., 1995, Hyman et al., 1995, Leverenz and Raskind, 1998). Directly related to mental retardation, a number of neuropathological changes found in DS CNS have been widely described, including reduced neuron number, alterations of cortical lamination, decreased dendritic ramifications and diminished synaptic spines, delayed myelination and reduce size of hemispheres (Golden and Hyman, 1994, Ross et al., 1984, Wisniewski, 1990). However, several studies indicate that astrocytes are also involved in DS, and display modifications of their number, structure and intracellular proteins (Fig. 2f).

### 7.1 Astrocytic over-population and structure

Despite the reduced number of neurons, which is due to severely impaired proliferation and increased apoptosis (Contestabile et al., 2007), DS brains present an altered number of glial cells, especially astrocytes. In particular, DS foetuses between 17 and 21 weeks of gestation have a higher percentage of cells with astrocytic phenotype and a smaller percentage of cells with neuronal phenotype in the hippocampus (Guidi et al., 2008), thus indicating a shift from neurogenesis to gliogenesis. Similar results were also found in the DS frontal lobe, where astrocytes were more numerous and morphologically more mature than those of age-matched controls (Zdaniuk et al., 2011). Furthermore, a two-fold developmental bias toward astrocyte differentiation in DS neural cultures (Briggs et al., 2013), which occurs through the release of astrocytic S100β (Chen et al., 2014), was reported using DS-induced pluripotent stem cells obtained by episomal reprogramming.

Astrocytes in DS are not only more proliferative and abundant, but they also display altered processes. DS brains display an impaired development of interlaminar astrocytes, which starts normally around 20–40 days after birth, but manifests a breakdown by the first year of age. This results in decreased number of interlaminar processes and immature astroglial layout, which can occur in several brain regions with different gravity (Colombo et al., 2005). Moreover, decreased GFAP mRNA levels have been found in the superior prefrontal cortex of DS subjects and in temporal lobe white matter of DS foetuses during middle pregnancy period (Goodison et al., 1993, Kanaumi et al., 2013). Similar results have also been obtained in the frontal lobe white matter of older patients. Interestingly, the pattern was reversed in the cortex, where an increase in GFAP^+^ cell number was observed, probably reflecting premature dementia-associated development of plaques and neurofibrillary tangles (Jørgensen et al., 1990, Mito and Becker, 1993).

### 7.2 Glutamate and Ca^2+^ signalling

Contradictory observations have been reported regarding the level of glutamate in DS: some post-mortem studies found no difference in glutamate or glutamine concentrations in the frontal lobes of foetal DS brains (Whittle et al., 2007), while others reported decreased levels in the hippocampus or unchanged levels in temporal and frontal lobe of adult DS subjects (Reynolds and Warner, 1988, Risser et al., 1997, Seidl et al., 2001). A more recent study found no significant difference in hippocampal glutamate-glutamine levels between adult DS subjects and controls (Tan et al., 2014), using *in vivo* proton magnetic resonance spectroscopy to overcome the limitations deriving from post-mortem analysis of neurotransmitters. This suggests that glutamate levels do not account for the cognitive impairment in DS.

However, an alteration in mGluR5 expression has been identified in patients with DS. White matter astrocytes of DS hippocampus display an increased prenatal mGluR5 expression, which persists post-natally. Furthermore, in adult DS patients with diffuse AD-associated neurodegeneration, higher levels of mGluR5 are also detected in astrocytes close to amyloid plaques (Iyer et al., 2014). The altered mGluR5 expression in astrocytes, which can affect intracellular Ca^2+^ signalling, is accompanied by augmented levels of S100β, as S100β protein exhibits a persistent over-expression in the hippocampus and in temporal, frontal and occipital lobes of DS foetuses, infants, children and adults (Griffin et al., 1998, Griffin et al., 1989, Jørgensen et al., 1990, Mito and Becker, 1993). Elevated levels of S100β mRNA have also been documented in the superior prefrontal cortex of adult DS brains (Goodison et al., 1993). Interestingly, a significant positive correlation has been found between S100β expression and patient age or cortical Aβ deposition, and the number of S100β-overexpressing activated astrocytes also strongly correlate with Aβ plaque density (Royston et al., 1999), thus suggesting a contribution of S100β overexpression to plaque formation and progression in DS.

The cognitive impairment observed in DS patients has been mainly attributed to defective neurogenesis and reduction in the number of neurons in several brain regions. However, the increased number of astrocytes found in DS brains, together with the altered morphology and the impaired expression of glutamate and Ca^2+^-signalling related proteins, suggest that a dysfunction of astrocytes may be a potential factor leading to DS intellectual disability.

## 8 Conclusions

During the last decades, the neurocentric view of the CNS has left the place to a multifaceted and more integrated paradigm of looking at and studying the brain. Nowadays, not only neurons but also other cell types and their reciprocal interactions are considered in explaining physiological CNS functions and dysfunctions. Human astrocytes display an intimate and dynamic structural-functional relationship with neurons, which makes them capable of regulating brain homeostasis and functioning. In the light of these physiological tasks, it is to be predicted that astrocytes can be implicated in cerebral pathology. Indeed, alterations of glial cells and neuroglial interactions have been widely described in animal models of various neurological diseases. Glial morphological and functional changes have been identified in post-mortem or surgically resected human brain tissue from patients with different brain disorders. In this review, we report evidence of astrocytic changes in the human brain in epilepsy, brain tumours, AD, MDD and DS. Prominent astrogliosis is a hallmark of epilepsy, brain tumours and AD. Remarkably, in these pathologies astrogliosis is associated with a decreased expression of astrocytic K^+^ channels and glutamate transporters, which leads to accumulation of K^+^ and glutamate in the extracellular space. MDD and DS instead show reduced extracellular glutamate levels and do not display reactive astrogliosis. In addition, a reduced number of astrocytes is observed in MDD, while an enhanced proliferation but impaired development of astrocytic populations occurs in DS.

Investigations conducted on human brain samples are particularly important to study the alterations leading to disease development and progression, since animal models are far from reproducing all the peculiarities of human pathologies. However, both the quantity of specimen and tissue are limited; furthermore, the access to proper control tissue is problematic, human testing being ethically inappropriate. Nonetheless, the study of human-specific changes in brain disorders becomes necessary to confirm findings obtained with animal studies and proceed towards the discovery of new therapeutic strategies; indeed, anatomo-pathological alterations do not always correlate with the clinical symptoms of the disease and animals do not develop the high cognitive functions which are specific of humans and that are lost during the progression of CNS diseases.

Targeting astrocytes thus represent an attractive alternative approach to develop new therapies to treat neurological disorders in humans. It has already been shown that targeting ephrin-A5, a molecule which is expressed by reactive astrocytes in the *peri*-infarct region, improves recovery after stroke in mice (Overman et al., 2012), and that currently used antidepressant drugs act by rescuing astrocytic defects, such as d-serine decrease (Malkesman et al., 2012). Moreover, there is evidence suggesting that a limitation of TNFα signalling specifically in astrocytes could be beneficial in autism spectrum disorders (Okabe et al., 2012, Petrelli et al., 2016, Yasui et al., 2013) and that some β-lactam antibiotics can increase GLT-1 expression and delay amyotrophic lateral sclerosis progression in animals (Rothstein et al., 2005). New therapeutic strategies based on transplantation of astrocytes or their progenitors are also promising, as indicated by the improved outcome observed in a mouse model of motor neuron disease (Lepore et al., 2008).

However, it is essential to better clarify which astrocytic alterations are pathologically relevant in different brain regions and whether disease-related glial changes are causative or simply an accompanying phenomenon. This will help build a more integrated picture of brain pathologies, which could result in novel effective therapeutic strategies targeting human astrocytes and their interactions with neighbouring neurons.

## Conflict of interest statement

The authors declare that there are no conflicts of interest.
